# SARS‐CoV‐2 Omicron variant: Characteristics and prevention

**DOI:** 10.1002/mco2.110

**Published:** 2021-12-16

**Authors:** Xuemei He, Weiqi Hong, Xiangyu Pan, Guangwen Lu, Xiawei Wei

**Affiliations:** ^1^ Laboratory of Aging Research and Cancer Drug Target State Key Laboratory of Biotherapy and Cancer Center National Clinical Research Center for Geriatrics West China Hospital Sichuan University Chengdu China; ^2^ West China Hospital Emergency Department State Key Laboratory of Biotherapy West China Hospital Sichuan University Chengdu China

## Abstract

Coronavirus disease 2019 (COVID‐19) has brought about a great threat to global public health. Recently, a new severe acute respiratory syndrome coronavirus 2 (SARS‐CoV‐2) variant B.1.1.529 has been reported in South Africa and induced a rapid increase in COVID‐19 cases. On November 24, 2021, B.1.1.529 named Omicron was designated as a variant under monitoring (VUM) by World Health Organization (WHO). Two days later, the Omicron variant was classified as a variant of concern (VOC). This variant harbors a high number of mutations, including 15 mutations in the receptor‐binding domain (RBD) of spike. The Omicron variant also shares several mutations with the previous VOC Alpha, Beta, and Gamma variants, which immediately raised global concerns about viral transmissibility, pathogenicity, and immune evasion. Here we described the discovery and characteristics of the Omicron variant, compared the mutations of the spike in the five VOCs, and further raised possible strategies to prevent and overcome the prevalence of the Omicron variant.

## Graphical abstract

Delta variant possesses the L452R and T478K mutations in the receptor‐binding domain (RBD), while 15 mutations have been accumulated in the RBD of the Omicron variant. Among these substitutions, a bunch of residues are located nearby the bound angiotensin‐converting enzyme 2 (ACE2) receptor.

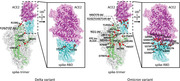

## 1 INTRODUCTION

The Coronavirus disease 2019 (COVID‐19) pandemic has been surging for almost two years. More than 260 million confirmed cases have been reported according to the statistics of the World Health Organization (WHO), including over five million deaths.^1^ The original severe acute respiratory syndrome coronavirus 2 (SARS‐CoV‐2) virus that was identified at the end of 2019 had evolved and a variety of variants emerged. In order to prioritize monitoring and research of these variants, WHO has classified them into three categories: variants of concern (VOCs), variants of interest (VOIs), and variants under monitoring (VUMs). The previous four VOCs include Alpha (B.1.1.7), Beta (B.1.351), Gamma (P.1), and Delta (B.1.617.2).^2^ They all resulted in a new wave of pandemic and thousands of deaths in more than one country and area, and even across the whole world. On November 26, 2021, a new variant named Omicron (B.1.1.529) was designated as the fifth VOC by WHO, which immediately raised global concerns.

## 2 EMERGENCE OF OMICRON VARIANT

According to the WHO reports, the first known confirmed infection by Omicron could be traced back to a specimen collected on November 9, 2021.^3^ The first Omicron sequence available, however, was from a specimen collected on November 11, 2021, in Botswana. Ever since the identification of Omicron, the variant appears to rapidly spread. A recent genomic‐sequence analysis on 77 virus samples collected in Gauteng province of South Africa from November 12 to 20 showed that all the analyzed variants were actually B.1.1.529,^4^ indicating that Omicron was becoming dominant in Gauteng. Furthermore, the identification of Omicron coincides with the recent sharp increase in the number of confirmed COVID‐19 cases in South Africa. The mean number of COVID‐19 cases per day increased from 280 to 800 after the Omicron variant was verified.^5^ This number exceeded 2000 on November 26, 2021, and broke through 10,000 on December 3, 2021.^6^ In addition, tracing the source of COVID‐19 cases revealed that B.1.1.529 had probably spread in western Europe before the first cases were detected in southern Africa.^7^

B.1.1.529 variant was first reported to WHO on November 24, 2021. On the day after receiving the report, WHO designated it as VUM and named it as Omicron variant (B.1.1.529). Only 2 days later, WHO categorized the Omicron variant as VOC, which recorded the shortest interval period of reclassifying a variant from VUM to VOC and subsequently brought about great public concerns. A few days after the identification of Omicron in Africa, the variant has emerged in the other continents. At the time of this writing, Omicron has been reported in 34 countries and areas, including Botswana, Hong Kong, South Africa, Israel, Belgium, Italy, and the USA.^8^ Apparently, the variant has not stopped spreading to other countries and regions.

Where and how the Omicron variant evolved remains to be investigated. The analysis of sequences of SARS‐CoV‐2 variants reveals that Omicron is a lot different from the other SARS‐CoV‐2 variants such that it is difficult to identify its closest relative.^9^ The results of phylogenetic studies indicate that the Omicron variant likely has diverged early from other SARS‐CoV‐2 variants rather than being developed from one of the previous VOCs.^9^ It is speculated that the Omicron variant might have been gestated in immunocompromised individuals (e.g., HIV patients co‐infected by SARS‐CoV‐2) for a certain period of time, or it might have evolved in a nonhuman species and is just recently spilled back into human beings.^10^

## 3 CHARACTERISTICS OF OMICRON VARIANT

**FIGURE 1:**
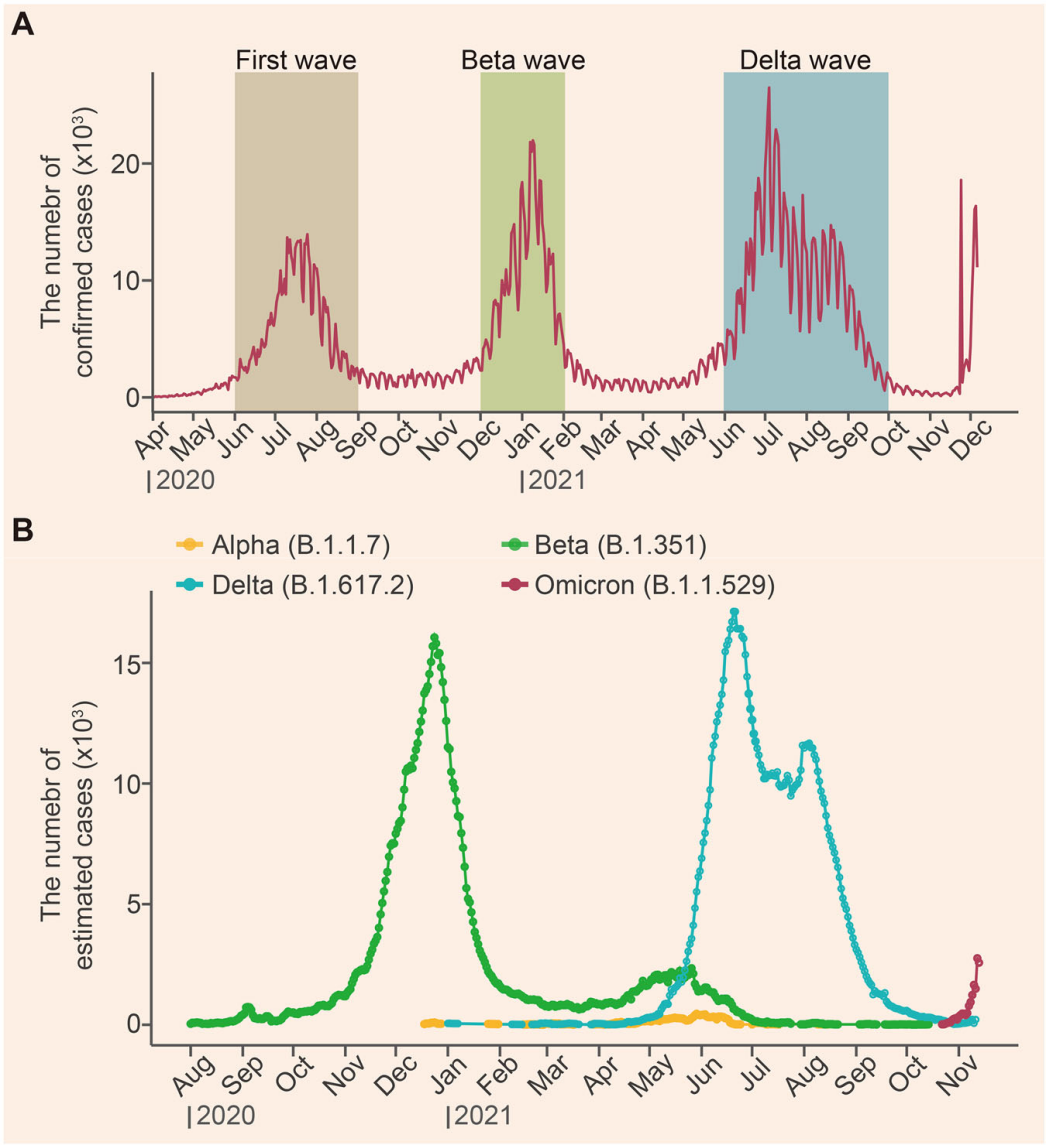
Waves of Coronavirus disease 2019 (COVID‐19) epidemics recorded thus far in South Africa. (A) Severe acute respiratory syndrome coronavirus 2 (SARS‐CoV‐2) has caused three waves of epidemics in South Africa. The number of daily confirmed infection cases is plotted. Data were downloaded from the World Health Organization (WHO). (B) The number of estimated cases infected by the indicated variants of concern (VOCs) in South Africa. The original data were downloaded from global initiative on sharing all influenza data (GISAID)

**FIGURE 2:**
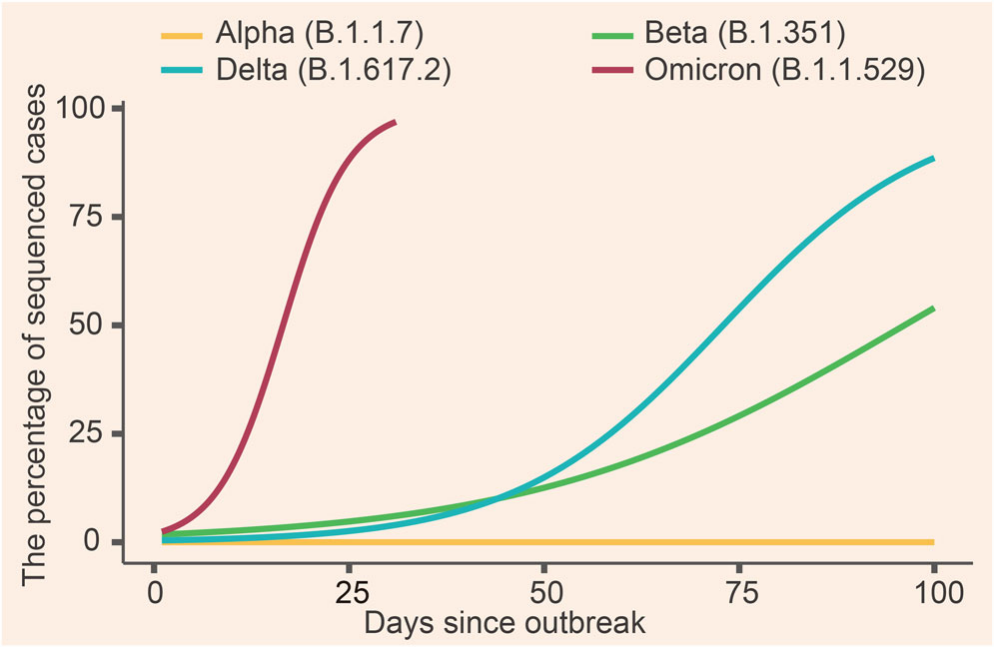
Omicron is spreading faster than other variants of concern (VOCs) in South Africa. The full genomic sequences were downloaded from global initiative on sharing all influenza data (GISAID), and the original data were processed with a logistic function

Since early 2020, three big waves of COVID‐19 outbreaks have been recorded in South Africa (Figure 1A). Among them, two are caused by the Beta and Delta variants respectively (Figure 1B). The epidemiological data showed that the percentage of infections associated with the Beta variant increased to ∼50% of the total daily infections within approximately 100 days since its outbreak (Figure 2). The infection percentage of the Delta variant, however, raised to ∼80% during the same period of time, echoing higher transmissibility among people for Delta than for the Beta variant. In contrast, the percentage of Omicron infection reached ∼90% within approximately 25 days in South Africa (Figure 2). The early doubling time of the Beta, Delta, and Omicron variants was calculated to be about 1.7, 1.5, and 1.2 days, respectively.^5^ These data indicate that the Omicron variant is probably more infectious than the Delta and Beta variants. It is also noteworthy that a recent retrospective study based on the population‐wide epidemiological data in South Africa indicates an increased risk of SARS‐CoV‐2 reinfection associated with Omicron.^11^ The possibility of a new wave of COVID‐19 epidemic in South Africa and even around the world therefore should not be ignored.

**FIGURE 3:**
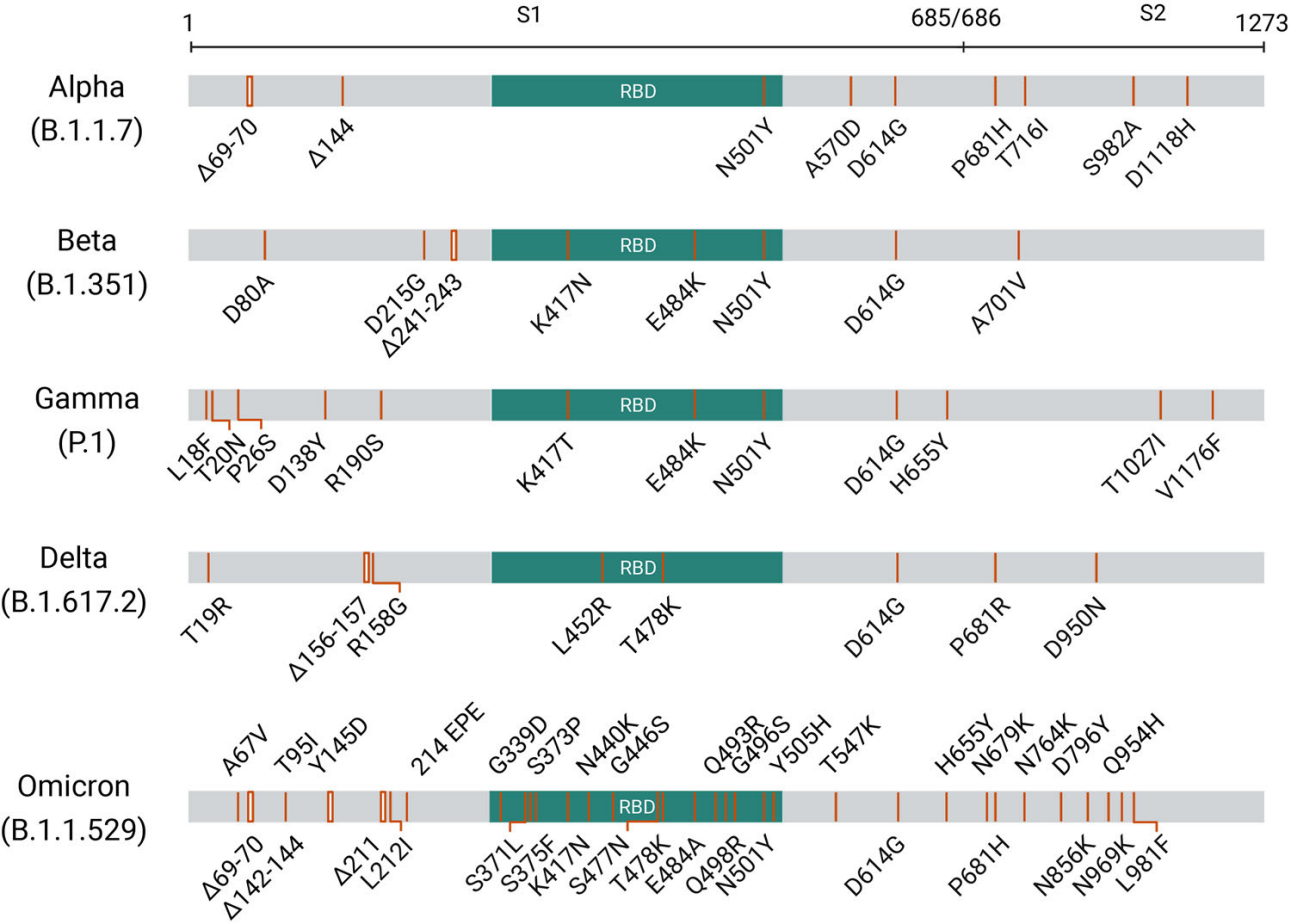
The schematic diagram showing the spike mutations of five variants of concern (VOCs). The mutations, including substitutions, deletions, and insertions, are defined based on the data from covariants at address https://covariants.org (20I for Alpha, 20H for Beta, 20J for Gamma, 21A for Delta, and 21K for Omicron, respectively)

Analysis of the genomic sequences of the Omicron variant has revealed a high number of non‐synonymous mutations, including several ones in spike that have been proved to be involved in transmissibility, disease severity, and immune escape. Overall, more than 60 substitutions/deletions/insertions have been identified in the Omicron variant,^12^ making Omicron a variant possessing the largest number of mutation sites of all SARS‐CoV‐2 variants characterized so far. Within ORF1a, the Omicron variant harbors six substitutions (K856R, L2084I, A2710T, T3255I, P3395H, and I3758V) and two deletions of in total four amino acids (amino acid 2083 and amino acids 3674–3676). Within ORF1b, the variant contains two substitutions (P314L and I1566V). In addition, a P10S substitution and a three‐residue deletion at positions 27–29 are observed in ORF9b. For the structural proteins, there are one substitution (T9I) in the envelope (E), three substitutions (D3G, Q19E, and A63T) in the membrane (M), and three substitutions and a three‐residue deletion in the nucleocapsid (N) proteins, respectively. While the aforementioned mutations emerge along the whole viral genome, the remaining mutations, which account for more than half of the total Omicron mutations identified, are accumulated in the spike. These include 30 substitutions of A67V, T95I, Y145D, L212I, G339D, S371L, S373P, S375F, K417N, N440K, G446S, S477N, T478K, E484A, Q493R, G496S, Q498R, N501Y, Y505H, T547K, D614G, H655Y, N679K, P681H, N764K, D796Y, N856K, Q954H, N969K, and L981F, three deletions of H69/V70, G142/V143/Y144 and N211, and one insertion of three amino acids (EPE) at position 214 (in some reports, the changes are described as the V143/Y144/Y145 deletion in combination with G142D and the L212 deletion in combination with N211I). In comparison to those observed in the other four VOC variants, the spike mutations identified in Omicron out‐number by about 3–4 times (Figure 3). It is notable that all the five VOCs contain the amino acid change D614G in spike. Previous studies have clarified that D614G is associated with higher upper respiratory tract viral loads and the younger age of patients.^13^, ^14^, ^15^ The Omicron variant also shares N501Y with the Alpha, Beta, and Gamma variants. This mutation is believed to enhance the binding between spike and angiotensin‐converting enzyme 2 (ACE2) and to induce higher transmissibility.^16^ When combined with the H69/V70 deletion, the transmissibility might be further increased.^17^ Besides, Omicron also has N679K and P681H mutations near the furin cleavage site. The incorporation of basic amino acids around the furin cleavage site could facilitate the cleavage of the spike into S1 and S2, thereby enhancing fusion and virus infection. As a matter of fact, the P681H mutation was also identified in the Alpha variant (Figure 3). This mutation has been suggested to enhance SARS‐CoV‐2 infectivity.^18^

**FIGURE 4:**
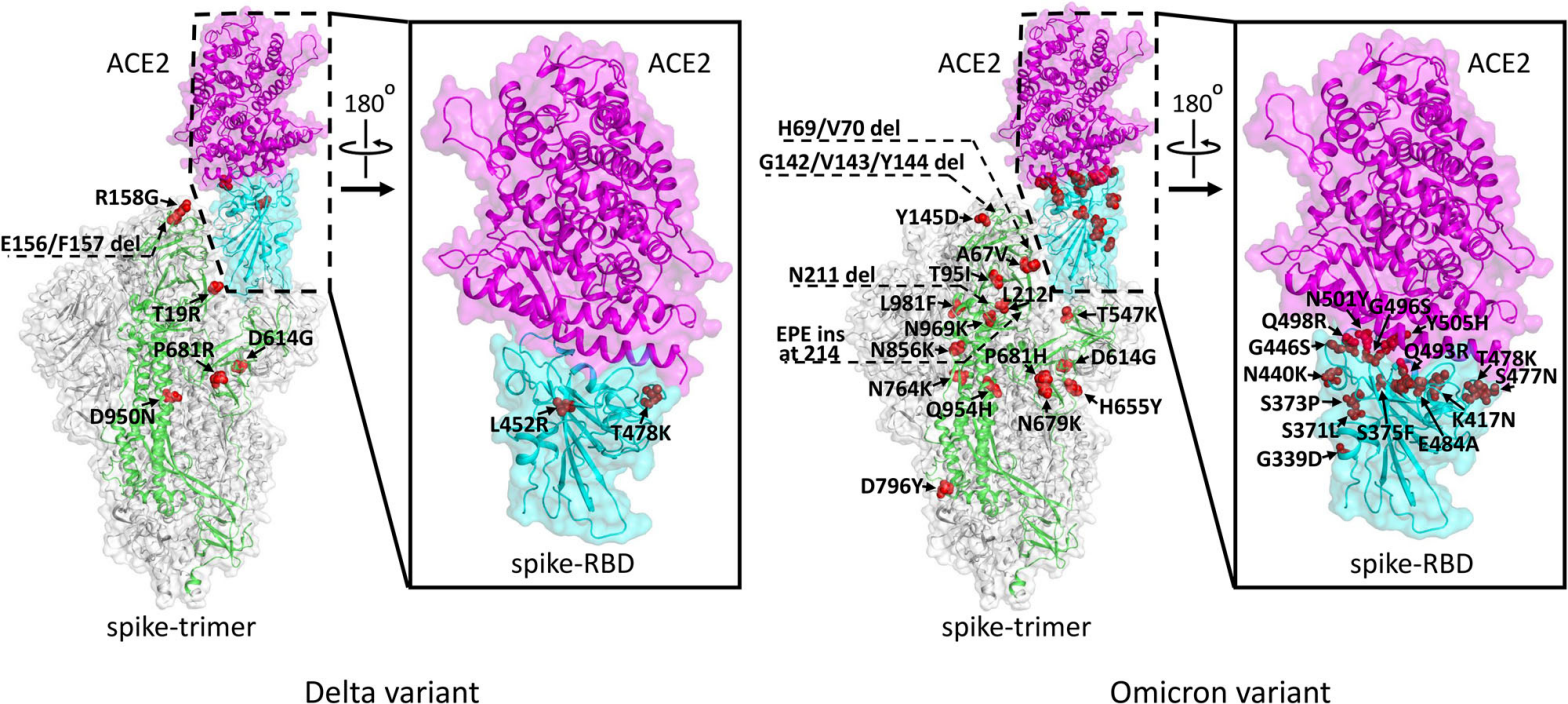
Landscape of spike mutations in the Delta (left) and Omicron (right) variants. The structures are depicted based on the cryo‐electron microscopy spike trimer structure of protein data bank (PDB) code 6VYB and the crystal RBD/ACE2 complex structure of PDB code 6LZG. One protomer of the spike trimer is highlighted in green and its receptor‐binding domain (RBD) in cyan. The bound angiotensin‐converting enzyme 2 (ACE2) receptor is colored magenta. The mutations defined based on covariants are labeled

It should also be noted that the spike receptor‐binding domain (RBD) is the authentic virus entity that recognizes the ACE2 receptor to mediate virus entry.^19^, ^20^ While the currently predominant Delta variant only possesses the L452R and T478K mutations in the RBD, 15 mutations have been accumulated in the RBD of the Omicron variant (Figure 4). Among these substitutions, a bunch of residues is observed to locate nearby the bound ACE2 receptor (Figure 4). How these mutations would affect the receptor binding, however, remains to be investigated in the future.

Whether or not and to what extent the Omicron variant can escape from immune recognition is another concern. It is notable that the spike RBD is the major target for neutralizing antibodies and that Omicron has accumulated 15 substitutions in the RBD region. We noted that multiple antigenic sites have been characterized in RBD, featured with the RBS‐A, RBS‐B, RBS‐C, the CR302, and the S309 sites.^21^ All the 15 mutations identified in Omicron spike RBD can be located to one or more of these antigenic sites, indicating potential resistance of Omicron to one or more of the monoclonal antibodies targeting these sites. As for the antibody treatment in clinical use, the cocktail consisting of LY‐CoV555 (also known as Bamlanivimab) and LY‐CoV016 (also known as Etesevimab) has been authorized for emergency use. Previous studies have revealed that the mutations at 484 and 417 positions of the spike are associated with immune evasion^22^ and that both Beta and Gamma variants could escape the neutralization of LY‐CoV555 (due to E484K) and LY‐CoV016 (due to K417N/T).^23^ Since the Omicron variant also contains E484A and K417N mutations, it is likely that Omicron would also resist these two antibodies.

Taken together, some spike mutations of Omicron have also been identified in the other VOC variants, such as D614G, N501Y, K417N, P681H, and the residue‐substitution of E484. These mutations have been indicated in higher binding affinity with ACE2, enhanced transmissibility and pathogenicity, and reduced ability of neutralization by monoclonal antibodies and immune evasion. The functions of other mutations and whether combined effects of these mutations exist, however, are not clear, resulting in great uncertainty about how the viral behavior and susceptibility will develop.

## 4 STRATEGIES FOR PREVENTION OF OMICRON VARIANT

### 4.1 Interruption of SARS‐CoV‐2 variant spread

At present, the detailed feature of the Omicron variant is unclear. In view of those spike mutations that are also observed in other VOCs, it is of particular concern that Omicron might have evolved with the capacity of easier spread among people and the ability to resist currently available antibody treatments. Such circumstance highlights the importance of maintaining present public health prevention measures, including wearing masks, frequent ventilation, keeping physical distance, and washing hands. These measures have been proved to be effective in interrupting the transmission of other variants and should also be effective in dealing with the Omicron variant. In addition, early diagnosis and timely quarantine are key factors that can minimize virus transmission during a pandemic. There have been epidemiological evidence showing that the failure of PCR tests by targeting the spike gene is rising along with the increasing cases infected by Omicron. Thus, improving diagnostic accuracy to enable timely isolation and treatment of diagnosed cases is also important to cut off the transmission of the Omicron variant.

### 4.2 Improving COVID‐19 vaccine coverage

Although some speculations show that the rapid spread of Omicron in South Africa might be a signal of a new wave of pandemic worldwide, the influence of this variant and what it means for the current pandemic are still far from clear. As a matter of fact, the situation of Omicron spread in South Africa could be dramatically different from those in other countries. For example, the proportion of the fully vaccinated population in South Africa is only about 24%.^24^ This value is far lower than the average vaccination percentage of 42% globally.^24^ This may accelerate the spread of Omicron in South Africa, highlighting the urgent need of increasing the vaccination coverage in the country. Although the authorized COVID‐19 vaccines showed decreased effectiveness against the variant viruses,^25^, ^26^, ^27^, ^28^, ^29^, ^30^, ^31^ it has been shown that the vaccines remain effective in preventing severe diseases, hospitalization, and death.^32^, ^33^, ^34^ It is also noteworthy that the low antibody level in the infected or the vaccinated against SARS‐CoV‐2 might promote the evolution and selection of new variants. In light of several studies reporting that the serum neutralizing antibodies dramatically decline 6 months post‐vaccination and that further vaccination with an extra booster dose can restore and even improve the vaccine effectiveness,^35^, ^36^, ^37^, ^38^, ^39^ we, therefore, believe that adding an extra boosting dose of the COVID‐19 vaccine to the vaccination program could undoubtedly help control the Omicron spread and infection.

### 4.3 Developing variant‐specific vaccines

It has been reported that the increased risk of SARS‐CoV‐2 reinfection is associated with the emergence of the Omicron variant in South Africa, indicating that the Omicron variant may be associated with substantial ability to evade immunity from prior infection.^11^ Moreover, whether the current COVID‐19 vaccines can protect against the Omicron variant attracts much attention. The most recent evidence showed that the current COVID‐19 vaccines provided less immunity to the omicron variant than other VOCs.^40^ Meanwhile, the sera from vaccinated individuals also had about 40 lower neutralizing ability against the Omicron variant compared to the wild‐type SARS‐CoV‐2.^41^ These results suggested that the present COVID‐19 vaccines might not be effective against the Omicron variant as other SARS‐CoV‐2 variants. More data about the effectiveness of current COVID‐19 vaccines need to be further investigated in the future.

Although the impact of the Omicron spike mutations on the effectiveness of currently available vaccines remains to be investigated, it is well documented that vaccines developed based on wild‐type SARS‐CoV‐2 are less effective in preventing variant infections.^31^ Our previous study has shown that the vaccine based on the mutant spike would have a higher level of neutralizing antibodies against mutant viruses, but lower neutralizing antibodies against wild‐type SARS‐CoV‐2.^42^ These observations highlight the importance of developing variant‐specific vaccines based on the mutated spike, especially towards the Omicron variant. Therefore, we are developing the specific vaccines against the SARS‐CoV‐2 Omicron variant based on the mutated spike of the Omicron variant. Alternatively, the vaccine candidates developed based on the other variants but containing one or more Omicron mutations might also be used to prevent the Omicron infection and transmission. For example, some unofficial information indicates that Moderna has developed two multivalent vaccine candidates: candidate mRNA‐1273.211 is believed to harbor several mutations observed in both the Omicron and Beta variants, and mRNA‐1273.213 is believed to have included a certain number of mutations present in the Omicron, Beta and Delta variants.^43^, ^44^ The effectiveness of these candidate vaccines against the Omicron variant needs to be further studied.

## 5 CONCLUSIONS

In face of the Omicron emergence, it remains an open question regarding the origin, the transmission capacity, and the immune‐escape potential of the variant. It is also not known if new variants might evolve on the basis of Omicron in the future. But there is no doubt that the Omicron variant will not be the last variant of SARS‐CoV‐2. The continuous emergence of new SARS‐CoV‐2 variants has made the control of the COVID‐19 pandemic more complicated. Fortunately, we have accumulated a lot of experiences and methods to deal with the novel coronavirus and we know what we need to do to stop the spread of virus variants. With global collaboration and rapid data sharing, human society would ultimately win the war against COVID‐19.

## CONFLICT OF INTEREST

The authors declare no conflict of interest.

## AUTHOR CONTRIBUTIONS

Xiawei Wei and Guangwen Lu conceived the study and revised the manuscript. Xuemei He wrote the paper. Weiqi Hong and Xiangyu Pan made the figures.

## ETHICS STATEMENT

Not applicable.

## Data Availability

The data included in this study are available upon request from the corresponding author.
